# Safety and efficacy of *C. muridarum* vaccines adjuvanted with CpG-1826 and four concentrations of Montanide-ISA-720-VG

**DOI:** 10.1038/s41541-024-00880-6

**Published:** 2024-06-10

**Authors:** Anatoli Slepenkin, Sukumar Pal, Amy Rasley, Matthew A. Coleman, Luis M. de la Maza

**Affiliations:** 1 Department of Pathology and Laboratory Medicine, Medical Sciences I, Room D440, University of California, Irvine, Irvine, CA 92697-4800 USA; 2 Physical and Life Sciences Directorate, Lawrence Livermore National Laboratory, Livermore, CA 94550-9234 USA; 3 University of California Davis, School of Medicine, Department of Radiation Oncology, Sacramento, CA 95616 USA

**Keywords:** Vaccines, Preclinical research

## Abstract

It is recommended that the adjuvant Montanide ISA 720 VG be used at a concentration of 70% v/v. At this concentration, Montanide causes at the site of immunization a local granuloma that can last for several weeks. To determine the safety and protective efficacy of a *Chlamydia muridarum* MOMP vaccine, formulated with CpG-1826 and four different concentrations of Montanide (70%, 50%, 30% and 10%), BALB/c (H-2^d^) female mice were immunized twice intramuscularly. Local reactogenicity was significant for vaccines formulated with 70% or 50% Montanide but not for those inoculated with 30% or 10% Montanide. Robust humoral and cell mediated memory immune responses were elicited by the 70%, 50% and 30% Montanide formulations. Mice were challenged intranasally with 10^4^ *C. muridarum* inclusion forming units (IFU). Based on changes in body weight, lungs’s weight and number of IFU recovered, mice vaccinated with the 70%, 50% and 30% Montanide formulations were significantly protected, but not mice receiving 10% Montanide. To conclude, we recommend the 30% Montanide concentration to be tested in humans and animal models to determine its safety and efficacy, in comparison to the 70% Montanide concentration currently used. The 30% Montanide formulation could significantly facilitate licensing of this adjuvant for human use.

## Introduction

It is estimated that each year approximatly 130 million individuals worldwide are infected in the genitourinary tract with *Chlamydia trachomatis*^1^. In addition, *C. trachomatis* causes ocular, respiratory, and gastrointestinal infections^2,3^. In females, genital infections can result in long-term sequelae including pelvic inflammatory disease, chronic abdominal pain, ectopic pregnancy and infertility^4,5^. Genital *C. trachomatis* infections are also associated with other serious diseases such as cervical hypertrophy, squamous metaplasia and HIV or HPV infections^6,7^. Babies born from mothers with a genital chlamydial infection can develop conjunctivitis and pneumonia with negative long-term medical sequelae^4^. Immunocompromised individuals can also have respiratory infections with *C. trachomatis*^8,9^. Antibiotic therapy is available, but due to the high proportion of asymptomatic patients, this approach has not eradicated these infections^10,11^. Furthermore, treated patients may fail to develop natural immunity that can result in an increase in the prevalence of *C. trachomatis* infections^11^. Therefore, *Chlamydia* infections are a worldwide public health problem and a vaccine is needed to control them^12–17^.

Over the last four decades several *Chlamydia* proteins have been tested as vaccine candidates.^12–23^. Among them, the major outer membrane protein (MOMP) is the only antigen that has completed a Phase I Clinical Trial^24^. MOMP is a homotrimer porin that accounts for 60% of the mass of the outer membrane of *Chlamydia* and has four variable domains (VD) and five constant domains (CD)^25–27^. The VD contain multiple B-cell epitopes while the T-cell epitopes are mainly located in the CD^28–30^.

Most subunit vaccines, including MOMP, lack the adjuvant activity necessary to induce robust innate and adaptive immune responses^31,32^. In the mouse model, it has been shown that both humoral and cell mediated immune responses contribute to protection against a *Chlamydia muridarum* challenge^33–35^. Therefore, to formulate a vaccine with MOMP we need to identify single adjuvants, or adjuvants combinations, that induce innate responses resulting in mucosal and systemic humoral and cellular immune memory. A combination of MOMP with 70% (v/v) Montanide ISA 720 VG, that elicits Th2 responses and CpG, that induces Th1 responses, has been found to be very effective at protecting mice against genital and respiratory chlamydial challenges, and non-human primates against ocular infections^15,20,36–39^. A shortcoming of this vaccine formulation is the induction of local reactogenicity at the site of immunization.

Early studies using Freund’s incomplete adjuvant (FIA), a water in oil formulation like 70% Montanide ISA 720 VG, resulted in injection site granulomas in humans and experimental animals^40^. Montanide ISA 720 VG is part of a large family of Montanide’s that were developed as an alternative to FIA to reduce its local reactogenicity and thus, the name Incomplete Seppic Adjuvant (ISA)^41,42^. Although the 70% concentration, is recommended by Seppic Inc., we could not find published data to determine the reasons for this recommendation. It is possible that this concentration was found by Seppic Inc., to elicit antibody levels similar or higher than those induced by FIA and to produce a long-term depot effect^41,43^. Montanide ISA 720 VG at the 70% concentration has been used in experimental animals and also in Phase I and II clinical trials with vaccine candidates to protect against HIV-1, HPV, SARS-CoV-2, malaria, leishmania and cancer^41,44–48^. In spite of many years of use, Montanide ISA 720 VG is not licensed probably because its significant reactogenicity at the site of immunization.

Montanide ISA 720 VG is an emulsion of squalene, a natural, vegetable, biodegradable, non-toxic, non-mineral oil, and a surfactant from the mannide monooleate family^43,49^. The mechanisms underlying the effects of this adjuvant are not well understood but elicits Th2-skewed responses. Vaccines containing squalene induce high antibody titers, numerous long-lived plasma cells, enhance specific cytotoxic T-lymphocyte responses, and stimulate the recruitment of antigen presenting cells (APC)^43,50^. When Montanide ISA 720 VG is used at a 70%/30% oil/water v/v ratio, the main activity is thought to be due to the depot effect that allows for the slow release of antigen at the site of immunization^43,49^.

CpGs (cytosine phosphoguanine motifs), agonists of TLR-9, elicit Th1-biased immune responses with direct activation of monocytes, macrophages and dendritic cells (DC) that secrete IL-6, IL-12, IFN-γ and TNF-α and several chemokines^51,52^. Furthermore, CpGs stimulate B-cells to proliferate and secrete immunoglobulins IL-6 and IL-12^53^. The overall effect of CpGs is the induction of strong Th1 humoral and cellular immune responses and broadening of the B-cell epitope recognition^51,52,54^. FDA has recently approved the use of CpG-1018, an adjuvant optimized for stimulating robust immune responses in humans, for a hepatitis B virus vaccine^55^.

To determine if it is possible to decrease the local reactogenicity while maintaining its adjuvanticity, here Montanide ISA 720 VG was tested at four different ratios (v/v) of the total vaccine, 70%, 50%, 30% and 10%. Amounts of CpG-1826 and MOMP were kept constant in the four formulations. We hypothesized that the vaccine that elicits the highest levels of IFN-γ from CD4 + T-cells will be the most protective but also the most reactogenic. The results showed that vaccine formulations containing 70%, 50% and 30% of Montanide ISA 720 VG, elicited equivalent immune responses and protection in mice. Furthermore, the 30% concentration induced minimal reactogenicity at the site of immunization. To our knowledge, this is the first time that four Montanide ISA 720 VG formulations have been tested in parallel for safety and efficacy in an animal model.

## Results

### Assessment of the reactogenicity at the site of immunization

**Fig. 1:**
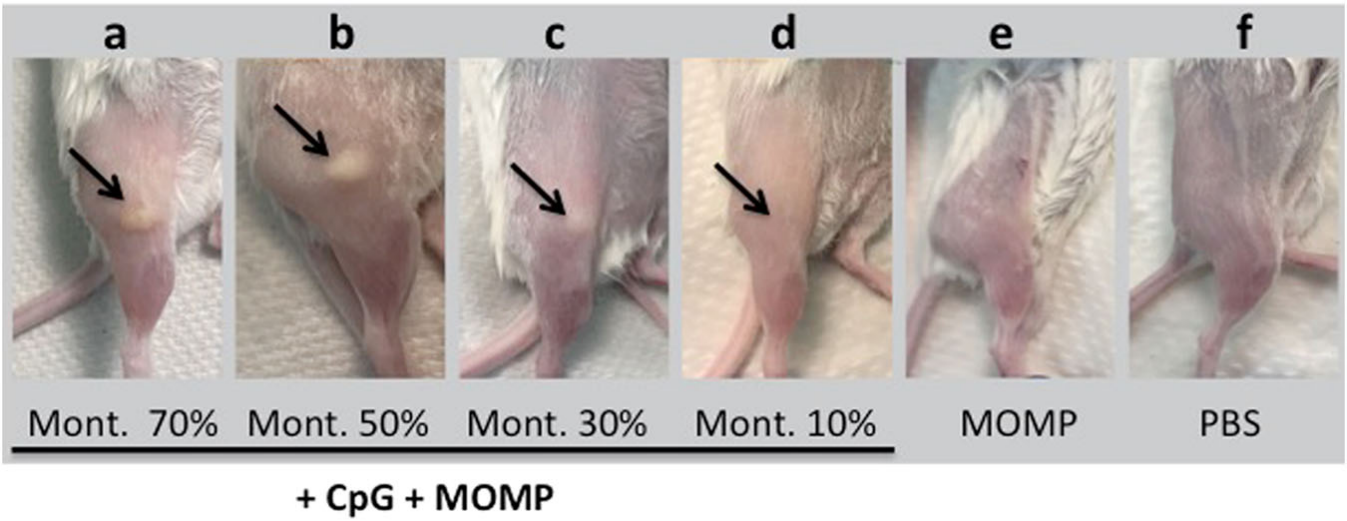
Evaluation of injection site reactogenicity. At euthanasia, pictures from representative mice were taken of the vaccination site. Mice immunized with Montanide ISA 720 VG: (**a**) 70%, (**b**) 50%, (**c**) 30% and (**d**) 10%; (**e**) MOMP without adjuvants and (**f**) PBS.

To evaluate the effects of the reactogenicity induced by the different vaccine formulations, mice were observed daily for a week after the two immunizations. No significant changes in the physical appearance or behaviour of the mice were observed. Pictures of the immunization site were taken at the end of the experiment. As shown in Fig. 1, in control mice that received PBS, or MOMP only, the site of immunization had a normal appearance. Similarly, no lesions were observed in mice immunized with 10% Montanide. In contrast, bullae ranging from ~ 2 – 5 mm in diameter were found in mice vaccinated with 70% and 50% Montanide while animals receiving 30% Montanide had no visible bullae, and only a few had indurations of ~ 1–2 mm in diameter.

### Antibody responses in serum and vaginal washes following vaccination

**Fig. 2:**
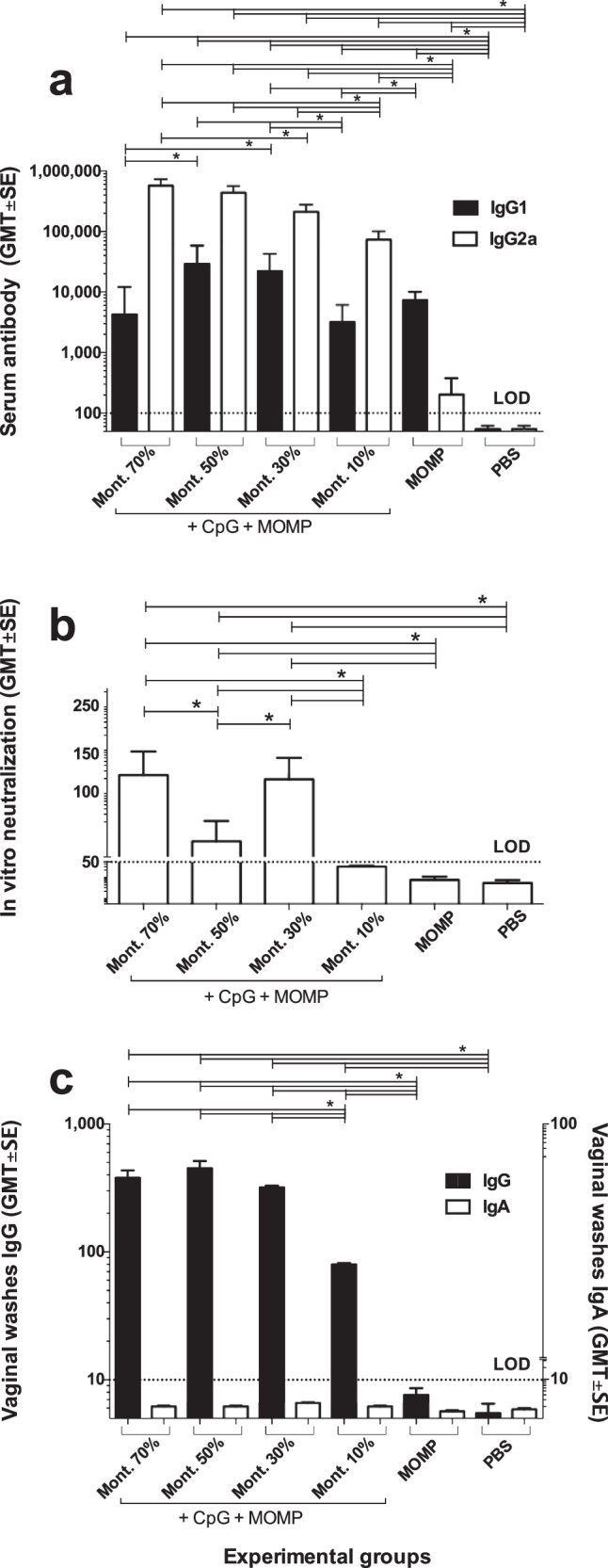
Antibody responses in serum and vaginal washes to *C. muridarum* EB following immunization. Mice were vaccinated and blood and vaginal washes from each animal were collected the day before the i.n. challenge. **a** IgG1 and IgG2a ELISA GMT in serum. **b** In vitro neutralizing antibody GMT in serum and (**c**) IgG and IgA antibody GMT in vaginal washes. LOD Limit of detection. *P < 0.05 by Student’s *t*-test.

Serum samples, collected the day before the challenge, were used to evaluate the type of immune responses elicited by vaccination. Sera collected before immunization were used as negative controls. Levels of IgG2a and IgG1 were determined by ELISA using *C. muridarum* EB as the antigen. As shown in Fig. 2a, the highest IgG2a GMT was observed in mice vaccinated using 70% Montanide, 517,547, while mice immunized with 10% Montanide had the lowest IgG2a GMT, 73,530. The mice with the highest IgG1 GMT were immunized with 50% Montanide, 29,372, while mice receiving 10% Montanide had the lowest IgG1 GMT, 3195. Based on the IgG2a/IgG1 ratio the four groups of mice immunized using adjuvants had Th1-biased immune response. Mice inoculated only with MOMP had a higher IgG1 GMT, 7342 than IgG2a, 202, indicating Th2-skewed responses. Controls receiving PBS had no detectable levels of antibodies to *C. muridarum* EB (Limit of detection < 100).

In vitro neutralization titers in serum were detected only in the three groups of mice immunized with 70% (119), 50% (59), or 30% (114) Montanide (Fig. 2b).

Mice vaccinated with Montanide had IgG GMT in vaginal washes ranging from 80–453 (Fig. 2c). Mice immunized with 10% Montanide had significantly lower IgG GMT than the other three groups (*P* < 0.05). The IgG GMT in vaginal washes for the negative controls immunized with MOMP only, or PBS were below the limits of detection (<10). Levels of IgA in vaginal washes were below the limit of detection for all groups of mice (<10).

**Fig. 3:**
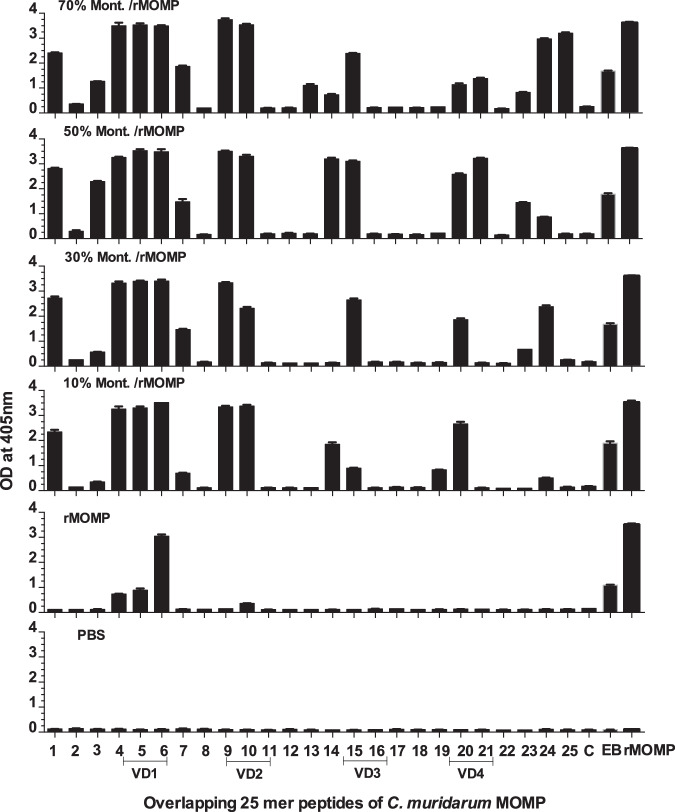
Binding of serum antibodies from immunized mice to synthetic *C. muridarum* MOMP peptides. Serum samples from vaccinated mice were collected the day before the i.n. challenge. Pooled serum samples from each group of mice were tested by an ELISA for their reactivity to 25-aa overlapping synthetic peptides corresponding to the *C. muridarum* mature MOMP. *C. muridarum* EB and MOMP were used as positive controls. C, a synthetic 25 mer non MOMP peptide, was used as negative control. Peptide 25, overlaps the N- and C- terminus of *C. muridarum* MOMP.

To determine the breadth of the antibody responses, 25 mer MOMP overlapping peptides were tested with sera using an ELISA. As shown in Fig. 3, the adjuvanted vaccines elicited antibodies to the four VDs, to peptides in CD1 and CD5 and to *C. muridarum* EB and MOMP. Antibody responses in mice immunized using 10% Montanide were weaker against peptides CD5 than in the other three groups. Mice immunized with MOMP had antibodies only to VD1, EB and MOMP. Sera from the negative control animals receiving PBS did not react with EB, MOMP or MOMP peptides.

### Cell mediated immune responses induced by vaccination

**Fig. 4:**
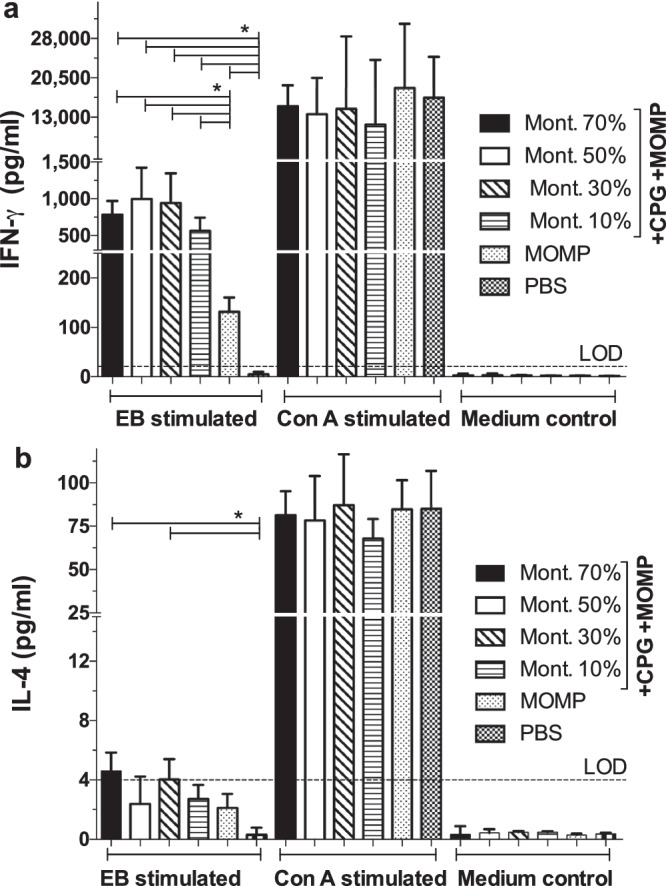
Determination of cytokines levels in T-cells supernatants collected from vaccinated mice the day before the i.n. challenge. Mice were immunized and the day before the intranasal challenge they were euthanized, their spleens collected, T-cells isolated using nylon wool columns and stimulated with *C. muridarum* EB, or with Concanavalin A as a non-specific stimulant, or with medium as a negative control. **a** IFN-γ levels in T-cell supernatants and (**b**) Levels of IL-4 in T-cell supernatants. Mice per group four. LOD Limit of detection. *P < 0.05 by Student’s *t*-test.

To evaluate the cellular memory immune responses, the day before the i.n. challenge, splenic T-cells were isolated using nylon wool from four mice/group and stimulated with *C. muridarum* EB. High levels of IFN-γ (pg/ml) were detected in the groups of mice vaccinated with 70% (781), 50% (996) and 30% (942) Montanide (Fig. 4a). T-cells from mice immunized with 10% Montanide secreted lower amounts of IFN-γ (565) (*P* < 0.05). Amounts of IFN-γ in mice immunized only with MOMP (131), or PBS (<20), were significantly lower than in the adjuvanted vaccines (*P* < 0.05). Levels of IL-4 in supernatants from T-cells stimulated with EB were low in all groups (Fig. 4b).

### Body weigh changes following the i.n. challenge with *C. muridarum*

**Fig. 5:**
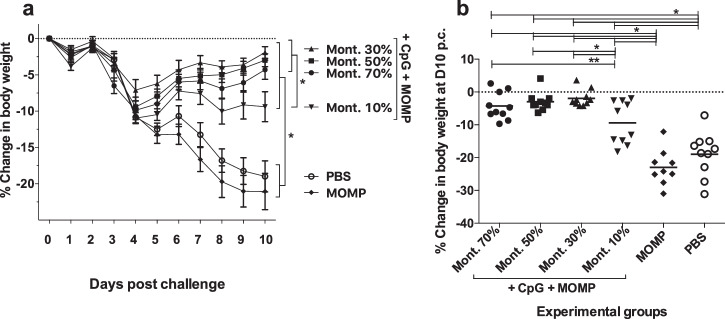
Daily changes in mean body weight following the i.n. challenge with 10^4^ *C. muridarum* IFU and changes in body weight at D10 p.c. Immunized mice were challenge i.n. with 10^4^ IFU of *C. muridarum* 4 weeks after the boost and changes in body weight recorded daily. At 10 days post-challenge mice were euthanized. Each group included 10 mice. **a** Percentage changes in daily mean body weight following the i.n. challenge with *C*. *muridarum*. *P < 0.05 by the Repeated Measures ANOVA. **b** % Changes in body weight at D10 p.c. *P < 0.05 by Student’s *t*-test. **P < 0.10 by Student’s *t*-test.

Four weeks after the boost mice were challenged i.n. with 10^4^ *C. muridarum* IFU and the body weight was determined daily for 10 days when all animals were euthanized (Fig. 5a). As determined by the repeated measures ANOVA test, all mice receiving adjuvants lost less body weight over the 10 d.p.c. when compared with the negative controls receiving MOMP only, or PBS (*P* < 0.05). All mice lost weight from D2 to D4 p.c. The two negative controls receiving PBS, or MOMP only, continued to lose weight until D10. Over the 10 days period, mice immunized with 10% Montanide lost more weight than the three other groups vaccinated using adjuvants (*P* < 0.05). Mice immunized with 30% Montanide recovered their body weight faster than the other three groups immunized with adjuvants.

At D10 post challenge, control mice receiving MOMP, or PBS, had lost 21.1% and 23.1% of their initial body weight respectively, significantly more than the weight losses of mice vaccinated with adjuvanted formulations (*P* < 0.05) (Fig. 5b and Table 1). At D10 p.c., mice immunized with 70%, 50%, 30% and 10% Montanide had lost 4.3%, 3.0% 1.9% and 9.4% respectively, of their initial body weight. Mice vaccinated using 50% and 30% Montanide lost significantly less body weight than those immunized with 10% (*P* < 0.05).

**Table 1:** Disease burden, yields of *C. muridarum* IFU and levels of IFN-γ and IgA in lungs’ supernatants at D10 post challenge

Vaccine	% Change body weight (mean ± 1 SE)	Lungs’ weight (g) (mean ± 1SE)	Median number IFU recovered from lungs (min-max) x10^3^	IFN-γ (pg/ml) (mean ± 1 SE)	IgA (OD_450_) (mean ± 1 SE)
MOMP+CpG/Montanide 70%	-4.3 ± 1.3^1,2,3^	0.23 ± 0.01^1,2,5^	14.3 (0.1–57,456)^7,8,9^	174 ± 102^1,2,3^	0.206 ± 0.03^1,2,4,6^
MOMP+CpG/Montanide 50%	-3.0 ± 0.9^1,2,4^	0.20 ± 0.01^1,2,4^	200.8 (0.3–2723)^7,8,9^	61 ± 11^1,2,4^	0.147 ± 0.02^1^
MOMP+CpG/Montanide 30%	-1.9 ± 0.8^1,2,4^	0.20 ± 0.02^1,2,4^	19.8 (0.9–2023)^7,8,9^	48 ± 1^1,2,4^	0.143 ± 0.01^1^
MOMP+CpG/Montanide 10%	-9.4 ± 2.1^1,2^	0.23 ± 0.01^1,2^	26,689 (17.2–132,125)^7,8^	628 ± 231^1,2^	0.120 ± 0.01
MOMP only	-21.1 ± 2.5	0.29 ± 0.01	423,185 (14,762–17,365,000)	2,194 ± 465	0.120 ± 0.02
PBS	-23.1 ± 2.8	0.28 ± 0.00	1,122,725 (114,950-4,884,000)	2,991 ± 393	0.096 ± 0.01

^1^*P* < 0.05 by the Student’s *t*-test compared to the PBS immunized mice.

^2^*P* < 0.05 by the Student’s *t*-test compared to the MOMP only immunized mice.

^3^*P* < 0.10 by the Student’s *t*-test compared to the MOMP+CpG-1826+Montanide 10% immunized mice.

^4^*P* < 0.05 by the Student’s *t-*test compared to the MOMP+CpG-1826+Montanide 10% immunized mice.

^5^*P* < 0.10 by the Student’s *t-*test compared to the MOMP+CpG-1826+Montanide 50% immunized mice.

^6^*P* < 0.10 by the Student’s *t-*test compared to the MOMP+CpG-1826+Montanide 30% immunized mice.

^7^*P* < 0.05 by the Mann–Whitney *U*-test compared to the PBS immunized mice.

^8^*P* < 0.05 by the Mann–Whitney *U-* test compared to the MOMP only immunized mice.

^9^*P* < 0.05 by the Mann–Whitney *U*-test compared to the MOMP+CpG-1826+Montanide10% immunized mice.

### Lung’s weights at D10 post *C. muridarum* challenge

**Fig. 6:**
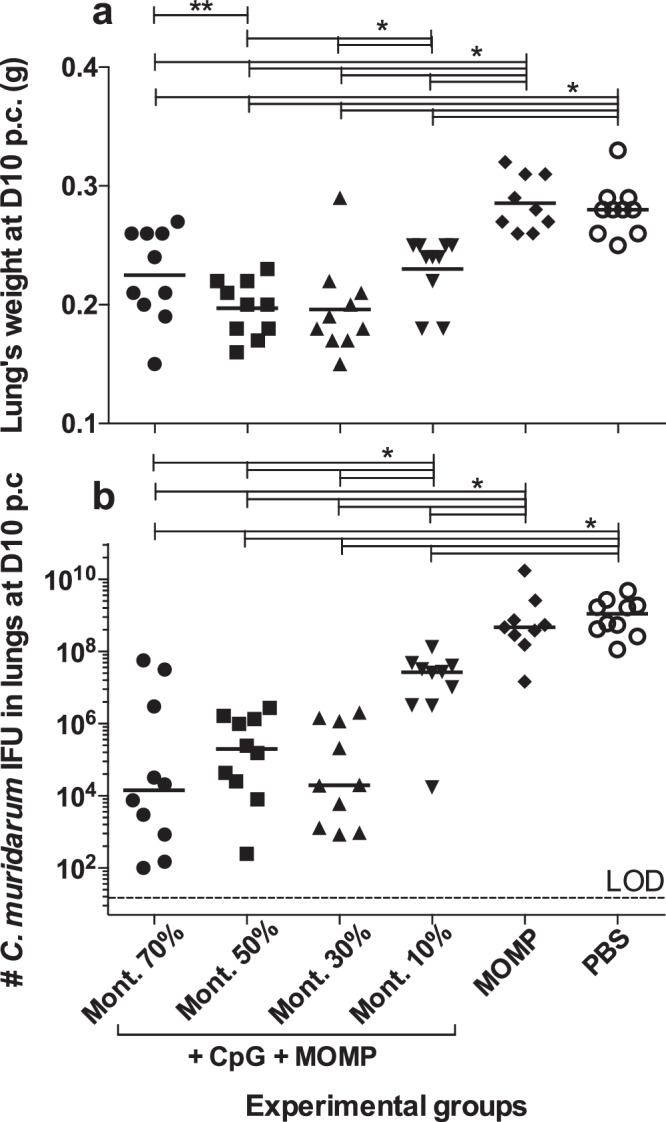
Determination of lungs’ weights and chlamydia burden at D10 following the i.n. challenge with 10^4^ *C. muridarum* IFU. Vaccinated mice were challenged i.n. with 10^4^ IFU of *C. muridarum*. At D10 p.c mice were euthanized, their lungs weighed, homogenized in 5 ml of SPG and cultured in HeLa-229 cell monolayers. Following staining with a monoclonal antibody to MOMP, the number of chlamydial inclusions was counted using light microscopy. Each group included 10 mice. **a** Lungs’ weights (g) at 10 days after the i.n. challenge. The mean is shown as a horizontal line. Each symbol represents an animal. *P < 0.05 by Student’s *t*-test. **P < 0.10 by Student’s *t*-test. **b** Number of *C. muridarum* IFU recovered from the lungs at D10 following the i.n. challenge. The median is shown as a horizontal line. Each symbol represents an animal. LOD Limit of detection. *P < 0.05 by the Mann–Whitney U-test.

As a parameter of the local inflammatory responses, the mean weights of the lungs (g) were determined following euthanasia (Fig. 6a and Table 1). Negative controls immunized with PBS, or MOMP only, had the heaviest lungs (0.28 and 0.29, respectively). These lungs’ weights were significantly heavier than those of the four groups receiving adjuvanted MOMP vaccines (*P* < 0.05). Mice vaccinated with 50% (0.20), or 30% Montanide (0.20) had lighter lungs than those immunized with 10% (0.23) (*P* < 0.05).

### Number of *C. muridarum* IFU recovered from the lungs at D10 p.c

The median number of *C. muridarum* IFU in mice, vaccinated only with MOMP, was 423,185 × 10^3^, while in mice immunized with PBS it was 1,122,725 × 10^3^ (Fig. 6b & Table 1). Both values were significantly different from the four groups of mice vaccinated using adjuvanted MOMP (*P* < 0.05). Mice vaccinated using 70% or 30% Montanide had the lowest median number of *C. muridarum* IFU in their lungs 14,300 and 19,800 respectively, while the group immunized with 10% had the highest median number of IFU 26,689 × 10^3^ (*P* < 0.05) among the groups receiving adjuvants. No statistically significant differences were observed in the number of IFU recovered from the lungs when comparing the three groups of mice immunized with 70%, 50% or 30% Montanide (*P* > 0.05).

### Levels of IFN-γ and *C. muridarum* specific IgA in lungs’ supernatants

**Fig. 7:**
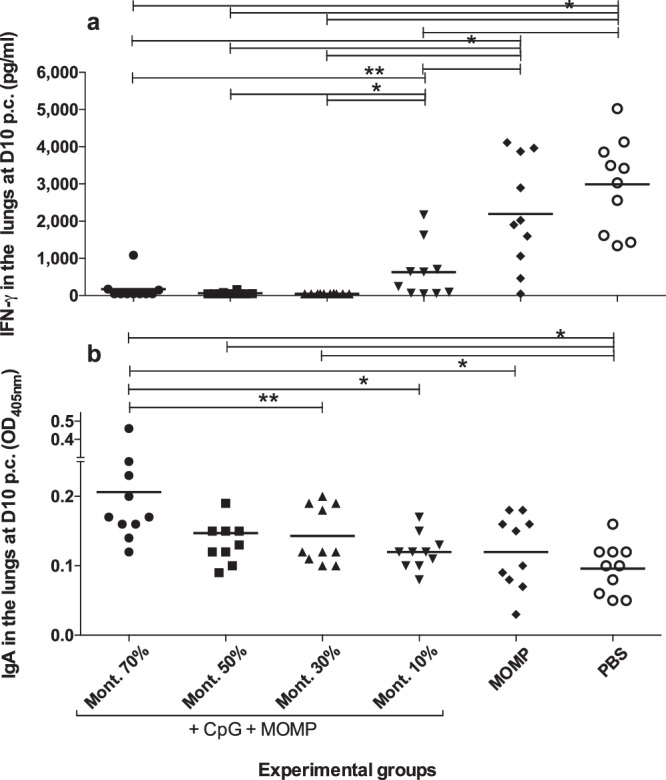
Immune responses in the lungs of mice at D10 p.c. Vaccinated mice were challenged i.n. 4 weeks after the boost with 10^4^ IFU of *C. muridarum*. At D10 p.c. the mice were euthanized, their lungs weighed and homogenized in 5 ml of SPG. Following homogenization, the lungs were centrifuged, the supernatants collected and used to determine the levels of IFN-γ and *C. muridarum* specific IgA. Each group included 10 mice. **a** IFN-γ levels in lungs’ supernatants at 10 d.p.c. The mean is shown as a horizontal line. Each symbol represents an animal. *P < 0.05 by Student’s *t*-test. **P < 0.1 by Student’s *t*-test. **b** *C. muridarum*-specific IgA levels in lungs’ supernatants at 10 d.p.c. The mean is shown as a horizontal line. Each symbol represents an animal. *P < 0.05 by Student’s *t*-test. **P < 0.1 by Student’s *t-*test.

It is expected that in mice that have cleared the infection, the mean levels of IFN-γ (pg/ml) in their lungs supernatants will be low, while in those that still have *C. muridarum* it will be high (Fig. 7a & Table 1). The negative controls immunized with MOMP alone, or PBS had high levels of IFN-γ, 2194 and 2991, respectively. Mice immunized with 70%, 50%, 30% or 10% Montanide, had significantly lower levels of IFN-γ, 174, 61, 48, and 628, respectively, than the negative controls (*P* < 0.05). Mice immunized with 10% Montanide had higher IFN-γ amounts than the other three groups that were vaccinated with MOMP plus the adjuvants (*P* < 0.05).

Levels of *C. muridarum*-specific IgA (OD_450_) were also determined in the lung’s supernatants. Mice that mount robust immune responses to vaccination are expected to have high levels of IgA. As shown in Fig. 7b and Table 1, the three groups of mice immunized with 70%, 50% and 30% Montanide had significantly higher levels of *C. muridarum* IgA 0.206, 0.147, and 0.143, respectively, than mice vaccinated with 10%, 0.120, or the two negative control groups receiving MOMP, 0.120 or PBS, 0.096 (*P* < 0.05).

## Discussion

The aim of this study was to compare the safety and efficacy of a vaccine formulated with MOMP, CpG-1826 and Montanide ISA 720 VG to induce in mice protective humoral and cell mediated immune responses against a *C. muridarum* respiratory challenge. Montanide ISA 720 VG was used at four different concentrations 70%, 50%, 30% and 10% (v/v) while the quantities of MOMP and CpG-1826 were kept constant in the four vaccines. When compared with the 30% and 10% concentrations, i.m. vaccination with the 70% and 50% formulations of Montanide ISA 720 VG produced higher reactogenicity at the site of immunization that lasted for the length of the experiment. Humoral and cellular immune memory responses were similar in mice vaccinated with the 70%, 50% and 30% Montanide ISA 720 VG but were weaker in the group immunized with 10%. Following the i.n. challenge, based on changes in body weight, weights of the lungs, and the number of C. *muridarum* IFU recovered from the lungs, vaccines containing 70%, 50% and 30% Montanide ISA 720 VG elicited similar robust protection while the 10% did not. Our results indicate that vaccines using 30% Montanide ISA 720 VG should be compared with the 70% formulation in humans and animal models for their safety and efficacy at inducing protection against pathogens.

The intensity of the reactogenicity of a vaccine is dependent on several factors including the site of immunization, volume of the vaccine, and type of antigen and adjuvant used^56^. The immunological status of the vaccinee will also impact the level of reactogenicity. When formulated as “a water-in-oil” emulsion (70% v/v), a shortcoming of Montanide ISA 720 VG is the production of a granuloma at the site of immunization that can last for weeks or months^43^. The Montanide ISA 720 VG induced granuloma, by creating a depot effect, helps to slowly release the antigen and maintain local immune responses over a long period of time. In addition to the depot effect, other mechanisms appear to be involved in the induction of immune responses by Montanide ISA 720 VG. For example, injection of the antigen in one site, and of Montanide ISA 720 VG at a different location, still has an adjuvant effect although it is weaker when compared with delivery at the same site^43^. Our results confirm that the depot effect of Montanide ISA 720 VG is only one of the components that affect its adjuvanticity. A similar phenomenon occurs with Alum. The depot effect is not necessary for induction of innate immune responses^57^. While the 70% formulation generated bullae, that measured ~ 2–5 mm in diameter, and were still present at the end of the experiment, the 30% Montanide ISA 720 VG formulation resulted in a ~1–2 mm indurations that disappeared over a period of 3–4 weeks. In spite of the differences in local reactogenicity between the Montanide ISA 720 VG at a 70% versus a 30% concentration both elicited the same immune responses indicative that they are independent of the depot effect. Attraction of APC to the immunization site could be a mechanism by which Alum and Montanide ISA 720 VG enhance immunity^43^.

Most studies in animal models, and data collected from patients, indicate that CD4 + T-cells producing IFN-γ, are required to protect against *C. trachomatis* infections, while CD8 + T-cells play a secondary role^34,58,59^. Although the role of antibodies in protection is still controversial, they are probably important particularly during the early stages of the infection^33,60,61^. For example, Brunham et al.^11^ demonstrated that levels of *C. trachomatis* secretory IgA specific antibodies in the cervix inversely correlated with the number of recoverable EB. Therefore, adjuvants combinations that elicit robust Th1 and a Th2 responses may be required for a chlamydia vaccine to optimize protection.

CpG-1826, delivered alone with MOMP, has a limited adjuvant effect likely because it readily diffuses systemically^62,63^. Enhancement of the immune responses has been observed when CpGs, or other TLR agonists, are delivered with Montanide ISA 720 VG^36,39,41,64^. These adjuvants combinations elicit more robust APC activation, resulting in enhanced expression of CCR7, MHC class II and co-stimulatory molecules that lead to strong T-cell activation in the lymph nodes^42,65^.

It is known that delivering antigens and adjuvants to the same APC helps to enhance immune responses^51,66^. The negative charges of MOMP likely interact with the positively charged polar head of cationic lipids on Montanide ISA 720 VG. CpG-1826 also carries negative electric charges and has strong electrostatic attraction to the surface of Montanide ISA 720 VG. The complex of MOMP+Montanide ISA 720+CpG-1826 can then bind to the positive charged surface of APC. As a result, colocalized delivery of antigen and adjuvants can enhance immune responses at the vaccination site^42,64,66–69^. Furthermore, by trapping multiple molecules, the adjuvant increases the density of the antigen leading to enhanced B-cell responses^67,70–72^.

Following immunization, the *C. muridarum* EB-specific lgG2a/IgG ratio in serum showed that the Montanide ISA 720 VG+CpG-1826 vaccines all elicited Th1-biased responses while MOMP, without adjuvants, induced Th2-skewed responses. Neutralizing antibodies in serum were present in the three groups of mice immunized with high concentrations of Montanide ISA 720 VG but were negative in the 10% group. These findings were supported by the high levels of IFN-γ present in the supernatants of T-cells stimulated with EB from mice vaccinated with 70%, 50% and 30% Montanide ISA 720 VG, but not with the 10% formulation.

Levels of protection against the i.n. challenge with *C. muridarum* paralleled the immune responses. Mice vaccinated with 70%, 50% or 30% Montanide ISA 720 VG lost less body weight than those immunized with the 10% Montanide ISA 720 VG. A similar trend was observed when the lungs’ weights of mice were determined at 10 days following i.n. challenge. Similarly, the number of *C. muridarum* IFU in the lungs were significantly higher in mice receiving the 10% Montanide ISA 720 VG than in the other three groups of mice. The high levels of *C. muridarum* specific of IFN-γ in T-cell supernatants, the presence of neutralizing antibodies in serum and of *C. muridarum* specific IgA in lung’s supernatants induced by formulations containing the 70%, 50% or 30% Montanide ISA 720 VG, likely are responsible for the protection observed against the respiratory challenge.

A limitation of this study is the use of the respiratory tract model rather than the genital tract model for infection with *C. muridarum*^73^. Testing for local and systemic reactogenicity is not a limitation since the i.m. route will likely be implemented when a vaccine for *C. trachomatis* genital infections becomes available. Furthermore, these results maybe can directly be applied to vaccines developed for respiratory bacterial and viral pathogens such as *Chlamydia pneumoniae*, *Mycobacterium tuberculosis*, *Streptococcus pneumoniae*, *Haemophilus influenzae*, *Bordetella pertussis*, influenza viruses, coronaviruses, and respiratory syncytial virus.

We have used the respiratory model extensively to test chlamydial antigens, adjuvants, and routes of immunization^16,23,74^. The respiratory and the genital tract have a mucosal and a systemic component and therefore, we can evaluate immune responses in both compartments. Immunization and effects on protection can be tested in the respiratory model in <3 months while experiments with the genital tract model take 7 months to complete. This is a major difference that significantly affects supplies and personnel costs. We have performed several experiments, and our conclusion is that, if we cannot induce protection in the respiratory model, that vaccine formulation and delivery system, likely will not protect against a genital tract challenge.

Another shortcoming of this experiment is that the immune responses and protective activity of these vaccines formulations were tested only 4 weeks following the boost. It will be important to test these vaccination protocols for their ability to induce long-term protection. It is possible that the 70% Montanide ISA 720 VG formulation, by having a longer depot effect, may induce extended memory immune responses when compared to the 30% Montanide ISA 720 VG vaccine. However, it is also possible that a long exposure to the antigen will result in tolerance with changes in the immune responses that will lead to increase susceptibility to infection^75^.

To summarize, for the first time, we have shown that a *C. muridarum* MOMP vaccine, formulated with 30% Montanide ISA 720 VG, combined with CpG-1826, elicits minimal local reactogenicity at the site of immunization, while inducing robust protective immune responses, similar to the 70% formulation, against a respiratory *C. muridarum* challenge. Our next step is to determine, in the genital tract mouse model, if the protective immune responses, elicited by the vaccine formulation containing 30% Montanide ISA 720 VG, induce an equivalent protection to that obtained with the 70% concentration. Studies in humans should then validate the results obtained with animal models. Positive data could help to move forwards the licensing of Montanide ISA 720 VG for clinical use.

## Methods

### Stocks of C. muridarum

*C. muridarum* (strain Nigg II; American Type Culture Collection) was grown in HeLa-229 cell using high glucose Dulbecco’s medium, plus cycloheximide (1 µg/ml) and gentamycin 10 µg/ml, without foetal bovine serum. Elementary bodies (EB) were purified and stored in sucrose phosphate glutamate buffer (SPG) at -80 °C as described^76^. The number of *C. muridarum* inclusion forming units (IFU) in the stock was assessed in HeLa-229 cells using immune-peroxidase staining with a *C. muridarum*-MOMP specific mAb (MoPn-40) produced in our laboratory^18^.

### Cloning, expression and purification of *C. muridarum* MOMP

The method to clone, express and purify the *C. muridarum* MOMP has been published^19^. By the Limulus amoebocyte assay (Associates of Cape Cod Inc.; East Falmouth, MA), MOMP had <0.05 EU of endotoxin/mg of protein.

### Vaccination of female BALB/c mice

Four-to-five-week-old female BALB/c (H-2^d^) mice (Charles River Laboratories) were vaccinated with *C. muridarum* MOMP (10 μg/mouse/immunization), twice at a 4 week interval by the intramuscular (i.m.) route in the quadriceps muscle. The following adjuvants combinations were used: CpG-1826 (Tri-Link) (10 µg/mouse/immunization) + Montanide ISA 720 VG (Seppic Inc.) at four different concentrations (70%, 50%; 30% and 10% v/v)^18^. Montanide ISA 720 VG was mixed with MOMP and CpG-1826 using a vortex (Fisher Scientific). Each formulation was vortexed for 1 min followed by 1 min rest at room temperature. The cycle was repeated five times. A negative immunization control received PBS, and an adjuvant negative control was injected only with MOMP. Mice were euthanized by injecting ketamine and xylazine followed by cervical dislocation. The University of California, Irvine, Animal Care and Use Committee (IACUC) approved the experimental vertebrate protocol.

### Evaluation of the humoral immune responses following immunization

Blood was collected from the periorbital plexus the day before vaccination and the day before the challenge. Using 96-well plates, ELISA antibody titers to *C. muridarum* EB (1 μg/well) were determined as described^21^. Goat anti-mouse IgG1 and IgG2a (BD Bioscience, San Diego, CA; Catalogue # 559626 and #553391, respectively) diluted 1:1000 for the two isotypes, were used. To stain, the substrate ABTS [2,2’-azino-bis-(3-ethylbenzthiazoline-6-sulfonate)] (Sigma-Aldrich, St. Louis, MO) was utilized and the plates were scanned in an ELISA reader at 405 nm. Titers were calculated using pre-immune sera ± 2 SD and reported as geometric mean titers (GMT). The limit of detection was 100.

The in vitro neutralization assays were performed in triplicate as described^77^. Two-fold serial dilutions of mouse serum were made in Ca^+2^/Mg^+2^ free PBS. Guinea pig serum (5%) was the source of complement. Samples were incubated with 10^4^ *C. muridarum* IFU for 45 min at 37 °C. Samples were then centrifuged onto HeLa-229 monolayers grown in flat bottom 96-well plates. Following incubation at 37 °C for 30 h in culture medium with cycloheximide (1 µg/ml), the cells were fixed with methanol. IFU were stained with mAb MoPn-40 and counted with a light microscope^77^. Neutralization was defined as greater than, or equal to, a 50% decrease in the number of IFU when compared with the controls incubated with pre-immunization serum^18^. The limit of detection was a titer of 50 or less.

Antibodies to linear epitopes induced by immunization were determined using overlapping 25 mers (SynBioSci Corp.; Livermore, CA), corresponding to the mature *C. muridarum* MOMP amino acid sequence^78,79^. Peptide 25 (p25) overlaps the N- and C-terminus of *C. muridarum* MOMP. EB and MOMP were used as positive controls and C, a non-MOMP 25-mer peptide, as negative control. Peptides were adsorbed onto high binding affinity ELISA microtiter plates (1 µg/well of a 96-well plate). Antibody binding was determined in triplicates using anti-mouse IgG as above^80^.

Humoral immune responses in the genital mucosa to *C. muridarum* EB, were assessed in vaginal washes collected the day before the i.n. challenge using samples collected before immunization as negative controls. Levels of IgG and IgA were determined as above in pooled samples. The limit of detection was 10 for IgG and IgA.

### Determination of cell mediated immune responses following immunization

Splenic T-cells, purified using nylon wool (>85% purity), collected the day before the challenge, from four mice/group, were stimulated with EB in the presence of irradiated (3300 rads, ^137^Cs) antigen presenting cells (APC)^81^. T-cells and APC were incubated in flat bottom 48-well plates (1.25 × 10^5^/well of each cell) at 37 °C for 48 h. with *C. muridarum* EB at a 1:1 ratio. Concanavalin A (5 μg/ml) was used as a positive stimulant, and cell culture medium (RPMI with 10% FBS) was the negative control. Quantities of IFN-γ and IL-4 in T-cells supernatants, were determined with commercial kits (BD Pharmingen, San Diego, CA)^21^.

### Intranasal challenge with *C. muridarum* and evaluation of the course of the infection in mice

Anesthetized mice were challenged i.n. with 10^4^ IFU of *C. muridarum* 4 weeks after the second immunization^82^. Daily body weight changes were assessed for 10 days post-challenge (d.p.c.) when mice were euthanized, their lungs weighed and homogenized (Seward Stomacher 80; Lab System) in 5 ml of SPG. To establish the number of *C. muridarum* IFU, six serial dilutions of the lungs’ homogenates were used to infect Hela-229 cells grown in 48 well plates. Following incubation for 30 h at 37 °C in a CO_2_ incubator, the IFU were visualized with mAb MoPn-40, and counted using a light microscope^81^. The limit of detection (LD) was < or less 50 *C. muridarum* IFU/lungs/mouse.

To evaluate the local cellular immune responses, quantities of IFN-γ in lungs’ supernatants at 10 d.p.c. were determined by an ELISA as described^63^. To assess the humoral immune responses in the lungs, levels of *C. muridarum* specific IgA were determined in the lung’s supernatants^63^. All animal experiments were replicated once.

### Statistical analyses

The two-tailed Student’s *t*-test was employed to evaluate differences between changes in body weight at day 10 post-challenge, lungs’ weights, levels of IFN-γ and IL-4 in T-cell recall assays, and levels of IFN-γ and IgA in lungs’ supernatants. Two-way repeated measures ANOVA with Sidak’s multiple comparison test was employed to compare changes in mean body weight over the 10 days of observation following the *C. muridarum* i.n. challenge. The Mann–Whitney *U-*Test was used to compare the antibody titers and the number of *C. muridarum* IFU in the lungs. A *P*-value of <0.05 was considered to be significant. A *P*-value of <0.1 was regarded as approaching significance. Statistical analyses were performed as discussed by Goodman^83^ and Rubin^84^.

### Reporting summary

Further information on research design is available in the Nature Research Reporting Summary linked to this article.

### Supplementary information

REPORTING SUMMARY

## Data Availability

All experimental data, related to this manuscript, will be provided upon reasonable request by the corresponding author.
